# Early Post‑Traumatic Osteoarthritis Features Greater Cartilage Extracellular Matrix Proteoglycan Loss Compared to the Pericellular Matrix

**DOI:** 10.1002/jor.70217

**Published:** 2026-05-10

**Authors:** Rasmus K. Hiltunen, Nina Pesonen, Simo P. Ojanen, Mikko A. J. Finnilä, Sanna Palosaari, Walter Herzog, Simo Saarakkala, Rami K. Korhonen, Juuso T. J. Honkanen, Petri Tanska

**Affiliations:** ^1^ Center of Oncology, Kuopio University Hospital Wellbeing Services County of North Savo Kuopio Finland; ^2^ Department of Technical Physics University of Eastern Finland Kuopio Finland; ^3^ Department of Medical Physics Hospital Nova of Central Finland, Wellbeing Services County of Central Finland Jyväskylä Finland; ^4^ Biocenter Oulu University of Oulu Oulu Finland; ^5^ Research Unit of Health Sciences and Technology University of Oulu Oulu Finland; ^6^ Research Unit of Translational Medicine, Faculty of Medicine University of Oulu Oulu Finland; ^7^ Medical Research Center Oulu University Hospital and University of Oulu Oulu Finland; ^8^ Human Performance Laboratory, Faculty of Kinesiology University of Calgary Calgary Alberta Canada

**Keywords:** cell mechanics, cell microenvironment, pericellular matrix, post‐traumatic osteoarthritis, proteoglycans

## Abstract

Proteoglycan loss in articular cartilage is a hallmark of post‐traumatic osteoarthritis, and it can affect both chondrocyte biomechanics and tissue health. Previously, we found that 4 weeks after anterior cruciate ligament transection (ACLT) in rabbits, proteoglycan loss was less pronounced in the pericellular matrix (PCM) than in the extracellular matrix (ECM), resulting in a greater PCM‐to‐ECM proteoglycan content ratio compared to controls. Here, we investigated whether this pattern is already present 2 weeks post‐ACLT and whether changes in the PCM‐to‐ECM proteoglycan content ratio are associated with chondrocyte volumetric strain in superficial cartilage during tissue loading. Unilateral ACLT was performed in eight skeletally mature rabbits. Two weeks post‐surgery, cartilage from load‐bearing sites of the operated and contralateral knees was collected. Age‐matched, non‐operated knees from separate animals served as controls. Proteoglycan content in the chondrocyte microenvironment was analyzed in the superficial and middle zones via digital densitometry. Volumetric strain of superficial zone chondrocytes was also measured using two‐photon confocal microscopy during compressive loading. In the superficial zone, the PCM‐to‐ECM proteoglycan content ratio was greater in the ACLT knees than in controls at the lateral tibial plateau (*p* = 0.043), but this was not clearly linked to changes in chondrocyte volumetric strain. In the middle zone, the PCM‐to‐ECM proteoglycan content ratio was greater in the ACLT knees than in controls for the medial femoral condyle, femoral groove, and patella (*p* < 0.05). These results indicate preferential ECM proteoglycan loss relative to the PCM in early post‐traumatic osteoarthritis, especially in the middle zone.

## 1 Introduction

Chondrocytes (cartilage cells) maintain and repair the extracellular matrix (ECM) of articular cartilage by converting mechanical stimuli into biochemical responses through mechanotransduction [1]. The ECM primarily consists of a fibrous collagen network, proteoglycans, interstitial fluid, and non‐collagenous proteins [2]. The highly organized collagen network provides tensile strength to the tissue, while proteoglycans in the interfibrillar space contribute significantly to compressive stiffness [3].

Osteoarthritis (OA) is the most common knee joint disease, and it significantly impacts patients' quality of life [4]. OA disrupts cartilage homeostasis, favoring catabolic processes [5]. Abnormal cartilage loading is a key contributor to OA development [6], triggering chondrocyte dysfunction and apoptosis [7]. Both excessive and insufficient cartilage loading can elevate the production of inflammatory and catabolic mediators, which suppress matrix synthesis and promote loss of proteoglycans and collagen [8]. Interestingly, early OA may also involve a localized healing response, enhancing proteoglycan production [9, 10] and potentially slowing down or even partially reversing matrix degradation [11, 12]. These local biomechanical and biochemical changes may influence how chondrocytes perceive and respond to mechanical signals [13, 14]. Therefore, understanding how the tissue matrix is altered and how those alterations shape mechanical signals (e.g., cell strains) in very early stages of OA is crucial for unraveling disease pathogenesis.

Chondrocytes are surrounded by the pericellular matrix (PCM), a specialized microdomain rich in collagen type VI and specific proteoglycans such as perlecan and decorin [14]. Together, the PCM and territorial ECM form a distinct microenvironment [15, 16]. Due to its proximity to chondrocytes, the PCM is thought to mediate both biomechanical and biochemical signals [17, 18]. Computational and experimental studies have suggested that proteoglycans in the PCM influence chondrocyte deformations and mechanotransduction [19, 20, 21, 22]. Although experimental data are limited, findings from animal models [23] and human studies [24] suggest that changes in pericellular proteoglycan and collagen content may precede alterations in the broader ECM.

Animal models help study OA pathogenesis in a controlled setting, minimizing variability found in human studies (e.g., age, sex, and weight) [25]. In rabbits, anterior cruciate ligament transection (ACLT) is commonly used to induce knee joint instability, quickly and reliably producing post‐traumatic OA with cartilage degradation patterns similar to those observed in humans [26]. In rabbits, site‐specific loss of ECM proteoglycan content can be detected 4 weeks post‐ACLT, accompanied by disorganization of the cartilage collagen network [27, 28]. More severe alterations, such as cartilage erosion and osteophyte formation, have also been reported 4 weeks post‐ACLT [29, 30].

Interestingly, the proteoglycan content within the chondrocyte microenvironment has been suggested to be less degraded than in the surrounding ECM, indicated as a greater PCM‐to‐ECM proteoglycan content ratio in operated knees compared to non‐operated controls [12]. We recently reported ECM proteoglycan loss as early as 2 weeks post‐ACLT while the collagen network remained largely intact [31], indicating less advanced cartilage degeneration than at 4 weeks post‐ACLT. Moreover, we detected changes in the superficial zone cell shape and volume change following mechanical loading of the tissue in operated knees compared to non‐operated control knees that we could not explain with the proteoglycan content changes in the ECM.

Thus, our primary aim was to determine whether changes in the proteoglycan content within the chondrocyte microenvironment in cartilage 2 weeks post‐ACLT, a time point selected to capture very early post‐traumatic OA, resemble those previously reported at 4 weeks post‐ACLT [12]. Specifically, we examined whether proteoglycan loss is relatively smaller in the PCM than in the surrounding ECM in the superficial and middle zones of cartilage, which would be indicated by a greater PCM‐to‐ECM content ratio. As mechanical stimuli in the ECM are transmitted through the PCM to chondrocytes, where they are converted to biochemical responses [17], the PCM may also modulate (and potentially amplify) strains perceived by chondrocytes [32]. Proteoglycan loss within the PCM has been linked to PCM softening [23], which may alter cellular strain sensing and influence chondrocyte volumetric strain under loading [19]. Such changes, for example, early after joint injury, may promote catabolic cellular responses and accelerate disease progression [23]. Therefore, as a secondary exploratory analysis, we examined whether superficial zone pericellular proteoglycan content is associated with chondrocyte volumetric strains previously quantified from the same sample set [31].

## 2 Methods

### 2.1 Animal Model

This study presents secondary analyses of earlier animal experiments [31, 33]. All proteoglycan content data reported in this study are new and unpublished. Cell volumetric strain results were included from our previous study [31]. Detailed descriptions of the experimental procedures are provided in Mustonen et al. [33].

**Figure 1:**
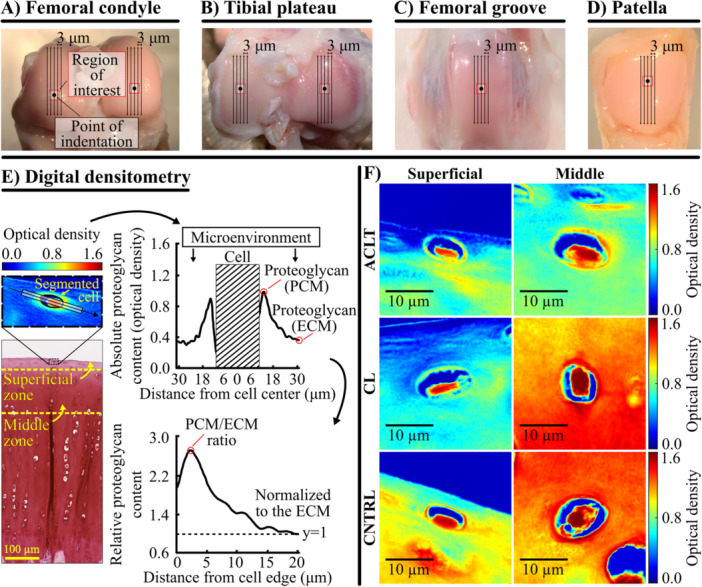
The knee joints of skeletally mature rabbits were harvested 2 weeks after anterior cruciate ligament transection surgery. Samples were prepared from the (A) lateral and medial femoral condyles, (B) lateral and medial tibial plateaus, (C) lateral femoral groove, and (D) patella. The primary load‐bearing areas of each cartilage surface were defined as regions of interest: the highest (proximal) points of the femoral condyles and the centers of tibial plateaus, femoral groove, and patella. (E) Histological sections were prepared as shown in subfigures A–D, and digital densitometry was used to analyze Safranin‐O‐stained histological sections for the proteoglycan content (optical density, scaled from 0 to 3.0) of the cell microenvironment in both superficial and middle zone cartilage. Proteoglycan content was analyzed from a rectangular region of interest extending 20 µm from the cell edge (height = 6 µm). The peak proteoglycan content value within 5 µm of the cell edge was designated as the pericellular matrix (PCM) value. The value 20 μm from the cell edge was set to represent the extracellular matrix (ECM). The PCM/ECM ratio was calculated to highlight changes in the cell microenvironment relative to the ECM. (F) Examples of optical density images acquired with digital densitometry from the femoral groove. Abbreviations: ACLT, anterior cruciate ligament transection; CL, contralateral; CNTRL, control.

Briefly, a unilateral ACLT surgery was performed in eight skeletally mature female New Zealand White rabbits (*Oryctolagus cuniculus*; age 12 months at the time of surgery; weight 4.44 ± 0.45 kg [mean ± standard deviation]). The operated knee joint was chosen randomly to avoid bias [31]. The rabbits were euthanized 2 weeks post‐surgery. Both operated (ACLT) and contralateral (CL) knee joints were harvested. Randomly selected knee joints, from either the left or right leg, from eight age‐matched and unoperated female rabbits were collected for the control group (CNTRL, weight 4.57 ± 0.35 kg). To reduce variability between experimental groups, only female rabbits were included. Osteochondral samples were then prepared from the lateral and medial femoral condyles, lateral and medial tibial plateaus, femoral groove, and patella (Figure 1A–D). All procedures were approved by the Committee on Animal Ethics at the University of Calgary and were carried out according to the committee's guidelines (ethical permit #AC11‐0035).

After tissue harvest, a simultaneous two‐photon confocal microscopy and in situ indentation testing [34] was performed in the main load‐bearing regions of cartilage under a force‐relaxation protocol to evaluate chondrocyte shape and volume before and after loading. Following the in situ indentation and confocal microscopy, reported in detail in our previous study [31] and briefly overviewed in Section 2.4, samples were fixed in formalin, decalcified, dehydrated in an ascending alcohol series, embedded in paraffin, and processed for polarized light microscopy and digital densitometry.

### 2.2 Polarized Light Microscopy and Digital Densitometry

Three unstained sections (5 µm thick) per sample were prepared for polarized light microscopy from the areas where the in situ indentation testing was performed. Polarized light microscopy was used to measure the average depth‐wise collagen orientation angle profile for the CNTRL group rabbits to define the superficial and middle zones at each cartilage location [31]. Furthermore, three Safranin‐O‐stained sections (3 μm thick) per sample were prepared from the same locations. The stained sections were imaged first with light microscopy to select superficial and middle zone chondrocytes for digital densitometry (Figure 1F). Chondrocytes that were visibly intact and located at least 30 µm from the nearest adjacent chondrocyte were chosen from the same areas where the indentation test was performed. Then, the proteoglycan content of the cell microenvironment (PCM and surrounding ECM) was estimated via digital densitometry, measuring the optical density of the Safranin‐O‐stained sections, using a previously established analysis protocol [12, 22, 35, 36]. This method has been originally validated with biochemistry [37, 38]. The number of cells imaged is shown in Supporting Information S1: Table S1. The measurement setup consisted of a light microscope (Microphot FXA, Nikon Co., Tokyo, Japan) connected to a CCD digital camera (ORCA‐ER, Hamamatsu Photonics K.K., Hamamatsu, Japan). Monochromatic light (*λ*: 492 ± 5 nm) and 40× magnification objective (NA: 0.7) were used to give a spatial resolution of 0.43 × 0.43 μm^2^. After collection, the resulting grayscale images (1024 × 1344 pixels) were converted into optical density images using a calibration line obtained with optical density filters (optical density values of air, 0.0, 0.3, 0.6, 1.0, 1.3, 1.6, 2.0, 2.3, 2.6, 3.0) and defined for each pixel of the CCD camera.

### 2.3 Analysis of the Chondrocyte Microenvironment

Chondrocytes were segmented by overlaying the digital densitometry images onto the light microscopy images (Supporting Information S1: Figure S1). Absolute proteoglycan content profiles of the chondrocyte microenvironment were averaged from a rectangular region (height: 6 μm) that extended 20 μm from the cell border towards the ECM on both sides of the cell (Figure 1E). The region was aligned parallel to the cell's major axis. The profiles were used to calculate the peak proteoglycan content value within 5 µm of each cell's edge, a region that presumably contains the PCM [39]. The peak value was used to describe the PCM proteoglycan content. Secondly, we calculated the PCM‐to‐ECM proteoglycan content ratio by dividing the peak proteoglycan content value by the value located at 20 μm from the cell's edge (ECM). This normalization provides a quantitative value for the changes in the cell microenvironment relative to the ECM. Lastly, the distance of the cell from the cartilage surface and the distance of the peak proteoglycan content from the cell edge were analyzed (Supporting Information S1: Tables S2,S3).

### 2.4 In Situ Two‐Photon Confocal Microscopy and Indentation Testing

**Figure 2:**
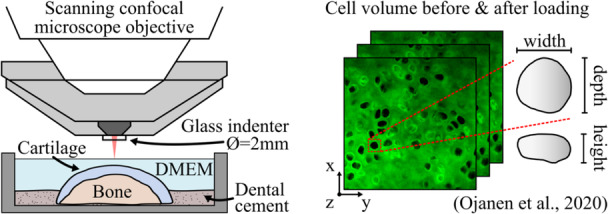
In our previous study [31], confocal microscopy imaging was performed on cartilage samples stained with dextran (matrix) and propidium iodide (dead cells) before and after force‐relaxation loading in indentation. Abbreviation: DMEM, Dulbecco's modified Eagle medium.

These measurements were previously performed in our earlier study [31]. Briefly, simultaneous two‐photon confocal microscopy and in situ indentation testing [34] were performed on the main load‐bearing regions of the cartilage after tissue harvest to determine how chondrocyte volume responds to loading, which is altered 2 weeks post‐ACLT surgery (Figure 2) [31]. Prior to the microscopy tests, the ECM and dead cells were stained with fluorescently conjugated dextran (D34682, Thermo Scientific, Eugene, Oregon, USA) and propidium iodide (P3566, Thermo Scientific, Eugene, Oregon, USA), respectively.

First, a pre‐load of 0.1–0.2 MPa was applied and maintained for 20 min, after which a “before loading” image stack was collected. The tissue was then indented at a rate of 10 µm/s until a load of 2 MPa was achieved. Subsequently, the indenter displacement was held constant for 20 min to allow full tissue relaxation, followed by acquisition of an “after loading” image stack at the same location. For each group and each location, approximately 40–70 cells were measured and analyzed using open source segmentation/analysis software pyCellAnalyst (http://pycellanalyst.readthedocs.io/en/latest/index.html). As measurements were performed on the intact cartilage surface, the cell volume analysis covered only the superficial zone of cartilage (depth penetration is limited to ~60–100 µm depending on the excitation wavelength, fluorophore, and laser power). Cell volumetric strain caused by the mechanical loading was analyzed as $\varepsilon_{\text{vol}}={(V}_{\text{after}}-V_{\text{before}})/\;V_{\text{before}}$, where *V*_before_ and *V*_after_ are the volumes measured before and after loading, respectively. Further details and complete results are presented in the Supporting Information: Section A, and in Ojanen et al. [31].

### 2.5 Statistical Analysis

A linear mixed‐effects (LME) model was used to compare the PCM‐to‐ECM proteoglycan content ratio and the peak proteoglycan content (located in the PCM) between the experimental groups. In the LME model, the experimental group, normalized depth of the cell, and side of the profile (“left” or “right”) were set as fixed effects. The samples were treated as a random effect to consider the within‐animal dependence among the operated rabbits. For pairwise comparisons, the least significant difference was used. Unless stated otherwise, results are presented as LME model estimated means ± 95% confidence intervals. Confidence intervals for percentage differences between the experimental groups were estimated using cluster bootstrapping with 5000 resamples. Statistical analyses were performed using IBM Statistics (version 29, IBM, Armonk, NY, USA) and MATLAB (R2023a, MathWorks Inc., Natick, MA, USA), with the level of significance set to *α* = 0.05.

## 3 Results

### 3.1 Pericellular Matrix to Extracellular Matrix Proteoglycan Content Ratio

**Figure 3:**
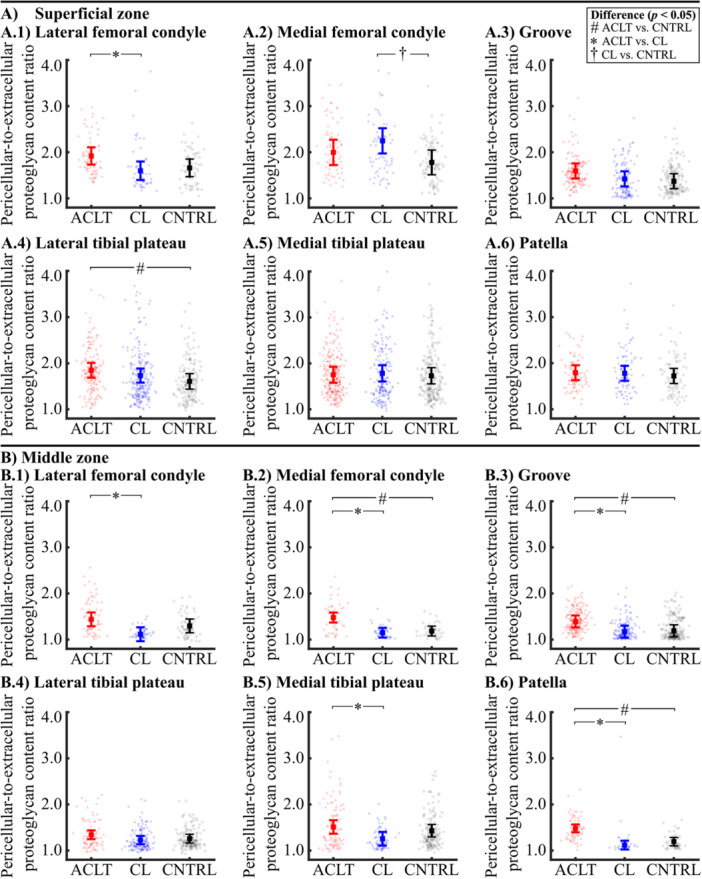
Pericellular‐to‐extracellular proteoglycan (PCM‐to‐ECM) content ratio of the (A) superficial and (B) middle zone cell microenvironment in the (1) lateral femoral condyle, (2) medial femoral condyle, (3) lateral femoral groove, (4) lateral tibial plateau, (5) medial tibial plateau, and (6) patella. The mean and 95% confidence intervals for each group are shown. Black points indicate the individual values. Abbreviations: ACLT, anterior cruciate ligament transection; CL, contralateral; CNTRL, control.

The PCM‐to‐ECM proteoglycan content ratio of the ACLT group knees was 15.0% (CI95: 2.6, 29.8%, *p *= 0.043) greater compared to the CNTRL group knees for the superficial zone cartilage of the lateral tibial plateau (Figure 3A.4). The PCM‐to‐ECM ratio in the lateral femoral condyle of the ACLT group knees was 20.1% (CI95: 6.5, 40.1%, *p *= 0.033) greater than that of the CL group knees (Figure 3A.1). In addition, the PCM‐to‐ECM ratio of the CL group knees was 26.3% (CI95: 9.4, 44.9%, *p *= 0.020) greater compared to the CNTRL group knees at the medial femoral condyle (Figure 3A.2).

In the middle zone cartilage, differences in the PCM‐to‐ECM proteoglycan content ratio between the ACLT and CNTRL group knees were more pronounced at several anatomical sites compared to the superficial zone (Figure 3B). The PCM‐to‐ECM ratio of the ACLT group knees was greater than in the CNTRL group knees at the medial femoral condyle (24.8% [CI95: 12.5, 36.6%], *p* < 0.001), the femoral groove (17.1% [CI95: 4.6, 30.5%], *p* = 0.033), and the patella (24.0%, [CI95: 14.5, 33.8%], *p* < 0.001). Additionally, compared to the CL group, the ACLT group knees exhibited 19.0%–32.4% greater PCM‐to‐ECM ratios at all anatomical sites except the lateral tibial plateau.

### 3.2 Peak Proteoglycan Content of the Cell Microenvironment

**Figure 4:**
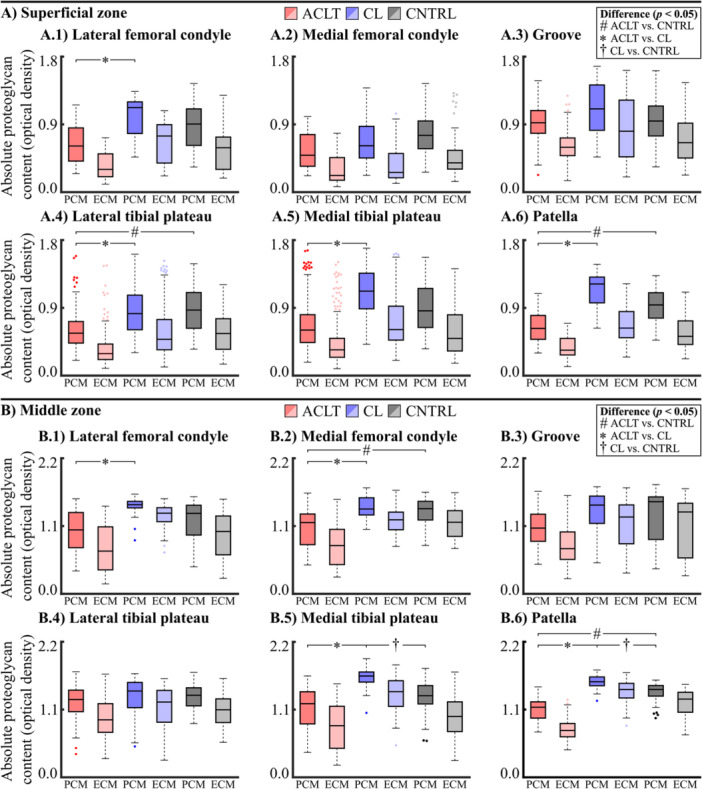
Box and whiskers plot for peak pericellular matrix (PCM, darker‐colored boxes) and extracellular matrix (ECM, dim‐colored boxes) proteoglycan content of the (A) superficial and (B) middle zone cell microenvironment in the (1) lateral femoral condyle, (2) medial femoral condyle, (3) lateral femoral groove, (4) lateral tibial plateau, (5) medial tibial plateau, and (6) patella. Circles represent outliers. Abbreviations: ACLT, anterior cruciate ligament transection; CL, contralateral; CNTRL, control.

**Figure 5:**
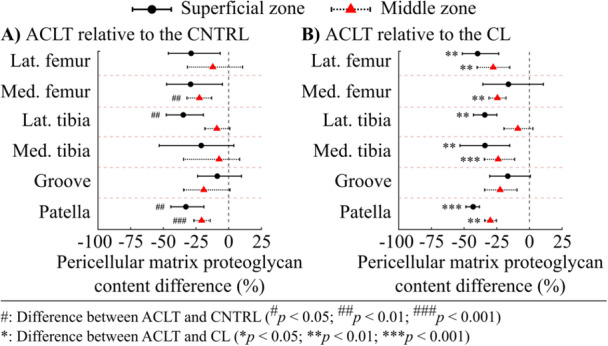
Comparison of absolute pericellular proteoglycan content between (A) anterior cruciate ligament transected (ACLT) knees and control (CNTRL) knees, and between (B) ACLT knees and contralateral (CL) knees. Data are shown separately for the superficial zone (black dots) and middle zone (red triangles). Error bars indicate bootstrapped 95% confidence intervals. See Supporting Information S1: Figure S3 for the corresponding results from the extracellular matrix.

In the superficial zone cartilage, the peak proteoglycan content (located in the PCM) was 34.6% [CI95: −47.6, −19.3%, *p* = 0.007] and 32.6% [CI95: −44.1, −18.9%, *p *= 0.005] smaller at the lateral tibial plateau and patella in the ACLT group compared to the CNTRL group (Figures 4, 5). Compared to the CL group, the ACLT group exhibited 33.9%–42.9% smaller peak proteoglycan content in the superficial zone cartilage of the lateral femoral condyle [−39.5%, CI95: −51.1, −23.4%, *p* = 0.008], the lateral tibial plateau [−34.0%, CI95: −42.6, −24.9%, *p* = 0.002], the medial tibial plateau [−33.9%, CI95: −52.9, −15.1%, *p* = 0.007], and the patella [−42.9%, CI95: −48.4, −38.1%, *p *< 0.001] (Figure 5B). The peak proteoglycan content did not differ between the CL and CNTRL groups at any of the anatomical locations in the superficial zone cartilage.

In the middle zone cartilage, the ACLT group exhibited 22.3% [CI95: −31.8, −13.0%, *p* = 0.003] and 20.6% [CI95: −26.6, −14.1%, *p *< 0.001] smaller peak proteoglycan content at the medial femoral condyle and patella compared to the CNTRL group (Figures 4, 5). Compared to the CL group, the ACLT group rabbits had 23.8%–29.8% smaller peak proteoglycan content in the middle zone cartilage of the lateral femoral condyle [−27.6%, CI95: −39.9, −15.0%, *p* = 0.008], the medial femoral condyle [−24.4%, CI95: −30.8, −17.8%, *p* = 0.001], the medial tibial plateau [−23.8%, CI95: −34.4, −11.3%, *p* < 0.001], and the patella [−29.8%, CI95: −34.1, −25.2%, *p *< 0.001] (Figure 5B). Interestingly, when comparing the CL group to the CNTRL group, the peak proteoglycan content in the middle zone cartilage was 21.8% [CI95: 13.8, 29.6%, *p* = 0.009] and 13.2% [CI95: 5.2, 21.8%, *p* = 0.010] greater in the medial tibial plateau and the patella.

Finally, the proteoglycan content in the PCM was generally reduced less than in the ECM for the ACLT compared to the CL or CNTRL group rabbits. This trend was observed in both the superficial and middle zone cartilages (Figure 5 and Supporting Information S1: Figure S3).

### 3.3 Relationship of PCM‐to‐ECM Proteoglycan Content Ratio, Pericellular Proteoglycan Content, and Chondrocyte Volumetric Strain in the Superficial Zone of Cartilage

**Figure 6:**
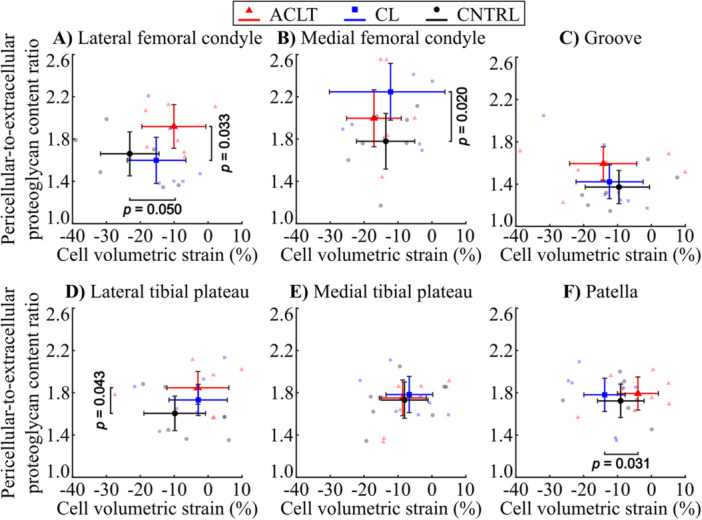
Pericellular‐to‐extracellular matrix proteoglycan content and cell volumetric strain in the superficial zone cartilage for (A) the lateral femoral condyle, (B) the medial femoral condyle, (C) the lateral femoral groove, (D) the lateral tibial plateau, (E) the medial tibial plateau, and (F) patella. The center markers (triangle, square, circle) represent mean sample‐wise values of pericellular proteoglycan content and volumetric cell strain (±95% CI) for anterior cruciate ligament transection (ACLT, red), contralateral (CL, blue), and the control (CNTRL, black) sample groups.

**Figure 7:**
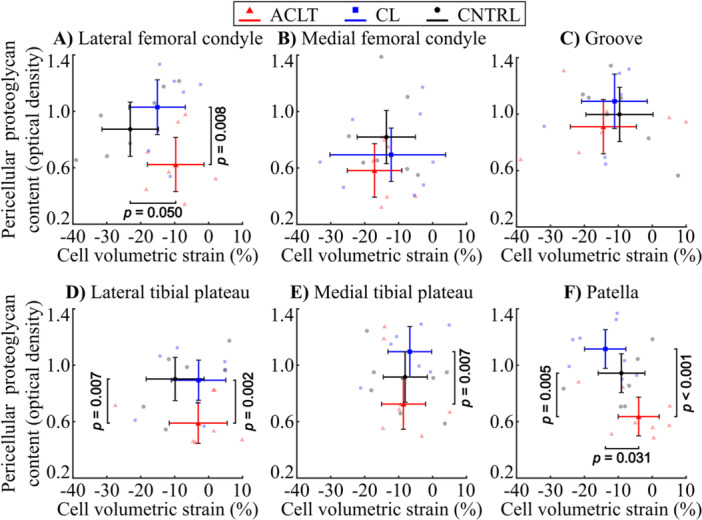
Peak proteoglycan content of the pericellular matrix and cell volumetric strain in the superficial zone cartilage for (A) the lateral femoral condyle, (B) the medial femoral condyle, (C) the lateral femoral groove, (D) the lateral tibial plateau, (E) the medial tibial plateau, and (F) patella. The center markers (triangle, square, circle) represent mean sample‐wise values of pericellular proteoglycan content and volumetric cell strain (±95% CI) for anterior cruciate ligament transection (ACLT, red), contralateral (CL, blue), and the control (CNTRL, black) sample groups.

In the lateral femoral condyle, chondrocytes from the ACLT knees experienced smaller compressive volumetric strain compared to those from the CNTRL group knees (*p *= 0.05, Figures 6A and 7A). Neither a higher PCM‐to‐ECM proteoglycan content ratio (*p* = 0.087) nor a smaller peak proteoglycan content of the PCM (*p* = 0.074) was able to explain the smaller deformation in the ACLT group knees. However, data points from the ACLT group formed a visually distinct cluster separate from both the CL and CNTRL groups in the lateral femoral condyle.

In the patella, chondrocytes from the ACLT knees experienced smaller compressive volumetric strain compared to the CL group (*p *= 0.031, Figures 6F and 7F) and had a smaller peak proteoglycan content in the PCM (*p* < 0.001). For the same location but compared to the CNTRL group, ACLT knee cartilage also had a smaller peak proteoglycan content in the PCM (*p* = 0.005) but no significant difference in compressive volumetric strain.

## 4 Discussion

We investigated the impact of very early post‐traumatic OA, at 2 weeks post‐ACLT surgery, on the proteoglycan content of the cell microenvironment in superficial and middle zone rabbit knee cartilages. Already at this 2‐week time point, the PCM‐to‐ECM proteoglycan content ratio was 17.1%–24.8% greater in the ACLT group compared to the CNTRL group in the middle zone cartilage for the medial femur, femoral groove, and patella. In the superficial zone, this greater PCM‐to‐ECM proteoglycan content ratio in the ACLT group compared to the CNTRL group was observed only in the lateral tibial plateau (15.0%). Together, these findings suggest preferential ECM proteoglycan loss relative to the PCM in early post‐traumatic OA, indicating that pericellular proteoglycan content is better preserved, most prominently in the middle zone of cartilage.

It has been suggested that the PCM is more resistant to proteoglycan degeneration than the ECM in early OA cartilage [12, 40]. The composition of the PCM is distinctly different compared to that of the ECM, and for example, collagen VI and proteoglycans, such as perlecan and biglycan, are expressed mainly in the PCM but not the ECM [41, 42, 43]. This unique composition of the PCM may explain why the proteoglycan loss was smaller and/or slower in the PCM in the early post‐traumatic phase of knee joint OA. For example, heparan sulfate, a key component of perlecan [44], has been reported to inhibit aggrecanase activity [45]. Additionally, biglycan, a small leucine‐rich proteoglycan [41], has been shown to have a higher resistance to proteolytic cleavage under inflammatory conditions compared to the main ECM proteoglycan, aggrecan [46].

Proteoglycan content in cartilage matrix represents the net balance between anabolic synthesis and catabolic degradation. Chondrocytes have also been reported to increase proteoglycan synthesis in early OA [47, 48]. Consistent with this, our gene expression analysis in the same animal model and time point [49] showed increased aggrecan (ACAN) expression in the lateral and trochlear compartments of ACLT knees compared with CNTRL knees. This anabolic response may indicate a possible matrix repair attempt by chondrocytes [50]. Despite this anabolic response, absolute proteoglycan content was generally smaller in the ACLT than in the CNTRL knees, indicating that proteoglycan levels were not maintained in the PCM (and were reduced even more in the surrounding ECM). Because the PCM neighbors the cell, any repair response should preferentially preserve PCM proteoglycans relative to the ECM. However, this repair response may have been insufficient to overcome a catabolic milieu, as supported by increased expression of IL‐6, MMP‐3, and MMP‐13 in ACLT cartilage [49].

The different magnitude of proteoglycan loss between the PCM and ECM could also relate to aggrecan retention. Recent studies have reported that decorin, a small proteoglycan, is pivotal to aggrecan retention in the ECM by strengthening the connection between aggrecan–aggrecan and aggrecan–collagen II molecules [51, 52]. Additionally, thinner type VI collagen fibrils in the PCM relative to the ECM may provide more space for decorin‐mediated interactions, potentially enhancing aggrecan retention in the PCM [53].

Overall, the ACLT group had smaller peak proteoglycan content in the PCM compared to the CL and CNTRL group cartilages in the superficial and middle zones. This reduction was more pronounced in the superficial zone cartilage (−32% to −43%) than in the middle zone cartilage (−20% to −30%). We observed similar findings in an earlier study on rabbit cartilage 4 weeks post‐ACLT surgery, where the peak proteoglycan loss in the PCM compared to the CL and CNTRL groups ranged from −28% to −40% in the superficial zone and from −14% to −26% in the middle zone [12]. This result also suggests that the loss of proteoglycan content in the PCM is of similar magnitude at 2 weeks and 4 weeks post‐ACLT. The observed variation in the proteoglycan content loss of the PCM in different cartilage zones might be due to the different capacity of catabolic activity within the cartilage. It has been suggested that the superficial zone of cartilage has higher levels of ADAMTS‐4, MMP‐3, and MMP‐13 during OA [54, 55]. Our gene expression analysis from bulk cartilage [49] indicated an upregulation of MMP‐3 and MMP‐13 in the ACLT group cartilage. Additionally, IL‐6, shown to increase ADAMTS‐4 expression [56], was also upregulated. This increased catabolic activity in the superficial compared to the middle zone cartilage might explain the difference in pericellular proteoglycan loss in these two regions.

The CL group had greater peak proteoglycan content in the PCM than the CNTRL group in the middle zone cartilage of the medial tibial plateau and patella. The finding in the medial tibial plateau might be explained by spontaneous degradation (moderately high OARSI score) observed in some CNTRL group rabbits [10]. Generally, differences in the peak proteoglycan content in the superficial and middle zone cartilage were more frequently observed between the ACLT and CL groups than between the ACLT and CNTRL groups. This result could be due to a natural adaptation of proteoglycan content caused by an unloading of the ACLT knees and increased loading of the CL knees [57].

We also investigated the influence of pericellular proteoglycan content on cell volumetric strain in native cartilage under mechanical loading for the superficial zone. In the patellar cartilage, the peak proteoglycan content of the PCM and the cell volume strain (i.e., volume loss) were smaller in the ACLT group than in the CL group. This result agrees with computational studies, which have shown that with greater PCM proteoglycan content (and greater stiffness), the two orders of magnitude softer cell [39, 58] compresses more under cartilage loading, leading to greater cell volume loss [19, 20, 21, 22]. However, such results were not observed for the other cartilage surfaces, where differences in PCM proteoglycan content or cell volume strain were observed. Although the proteoglycan content of the PCM influences pericellular stiffness [23, 39, 59] and chondrocyte deformation under cartilage loading [19, 20], it is unlikely to fully explain early changes in cell volumetric strain in post‐traumatic OA. Since axial cartilage strain during loading did not differ between groups [31], and differences in ECM proteoglycan content did not account for the smaller volumetric cell strain in the ACLT than the CNTRL and CL groups [31], factors other than decreased proteoglycan content must have contributed to the smaller volumetric cell strain in the ACLT group. One likely factor could be the disorganization or enzymatic cleavage of collagen fibrils near the superficial zone chondrocytes. The organization of collagen fibrils is known to influence the mechanical behavior of both the tissue and chondrocytes, as shown by experimental and computational studies [19, 22, 60, 61], and disorganization of collagen fibrils has been suggested to occur at an early stage of OA, before the loss of collagen in articular cartilage [62, 63].

This study has some limitations. The sample groups (8 operated, 8 control rabbits) are small, thus limiting statistical power. Additionally, the proteoglycan content was not analyzed from the same cells as the cell deformation [31]. To account for this difference, cells (*n* = 11 per zone from each location and sample) were analyzed via digital densitometry from the same region where the deformation analysis was done. Also, the proteoglycan content was indirectly estimated from Safranin‐O‐stained sections. Safranin‐O binds stoichiometrically to the negative charges of the glycosaminoglycan chains of proteoglycans [37]. Thus, the measured optical density reflects fixed charge density in cartilage and provides a surrogate measure for spatial proteoglycan content [38]. From the sections, the PCM was identified based on peak Safranin‐O‐staining intensity within close proximity of the cell rather than, for example, collagen VI immunolabeling [64]. During OA, the shape of the PCM and the proteoglycan distribution within the PCM can vary [14, 65]. For example, in this study, the peak proteoglycan content was localized closer to the cell edge in the ACLT group knees than in the CL and CNTRL groups (Supporting Information S: Table S2). Additionally, the PCM integrity might become compromised during disease [66] if the various regulatory matrix molecules within the PCM degrade [67]. These changes may reduce the accuracy of our approach of localizing the PCM. However, the intense Safranin‐O‐staining of the PCM has been shown to localize with collagen VI in the PCM [15], and we restricted our analysis to visually intact chondrocytes. Thus, peak Safranin‐O intensity was deemed feasible to identify the PCM for this study. Finally, the surgical transection of the ACL in the rabbit model does not perfectly mimic the uncontrolled ACL rupture in humans caused by (often sporting) accidents. However, changes in the physical properties of cartilage between surgical ACLT and injury‐induced tearing of the ACL have been shown to be similar [26]. Naturally, the small but distinct differences between human and rabbit knee joint shapes, cartilage structure, and cartilage biomechanics must be kept in mind when translating our results to those associated with human ACL tears caused by accidents.

We used the time point 2 weeks post‐ACLT to study the early effects of post‐traumatic OA in the PCM. As the proteoglycan loss in the PCM was of similar magnitude to that observed at 4 weeks post‐ACLT, future studies should examine even earlier time points to better capture disease onset and initial progression. Based on the results of this study, we conclude that 2 weeks post‐ACLT, the PCM proteoglycan content was relatively better preserved than the ECM proteoglycan content, reflected by an increased PCM‐to‐ECM proteoglycan ratio. This pattern was more pronounced in the middle zone than in the superficial zone. Finally, superficial zone PCM‐to‐ECM proteoglycan content ratio changes were not clearly associated with the observed reductions in chondrocyte volumetric strains during compressive tissue loading.

## Author Contributions

**Rasmus K. Hiltunen:** conceptualization, methodology, software, formal analysis, investigation, writing – original draft, writing – review and editing, visualization, funding acquisition. **Nina Pesonen:** conceptualization, software, formal analysis, investigation, writing – review and editing. **Simo P. Ojanen:** conceptualization, methodology, investigation, formal analysis, writing – review and editing, funding acquisition. **Mikko A. J. Finnilä:** conceptualization, investigation, methodology, writing – review and editing. **Sanna Palosaari:** investigation, writing – review and editing. **Walter Herzog:** conceptualization, methodology, funding acquisition, writing – review and editing. **Simo Saarakkala:** conceptualization, methodology, funding acquisition, writing – review and editing. **Rami K. Korhonen:** conceptualization, methodology, funding acquisition, writing – review and editing. **Juuso T. J. Honkanen:** conceptualization, funding acquisition, writing – review and editing. **Petri Tanska:** conceptualization, methodology, funding acquisition, writing – review and editing.

## Conflicts of Interest

The authors declare no conflicts of interest.

## Supporting information

Supporting File

## Data Availability

The data that support the findings of this study are available from the corresponding author upon reasonable request.
